# Research on Multifeature Intelligent Correction of Spoken English

**DOI:** 10.1155/2022/8241241

**Published:** 2022-01-27

**Authors:** Yanyan Luo

**Affiliations:** School of Foreign Languages, Hefei Normal University, Hefei 230601, China

## Abstract

For a long time, college English teaching in many Chinese universities has focused on cultivating students' reading abilities while ignoring the cultivation of students' speaking abilities, leaving many non-English majors unable to communicate in English even after years of English study. This paper outlines the entire design and development process for an intelligent correction system for spoken English, with a focus on the methods for implementing the functions of spoken English examination, question bank management, and marking. A multifeature fusion of SE (sample entropy) and MFCC (Mel frequency cepstrum coefficient) based speech emotion recognition method is proposed. It denotes the rate at which the SE nonlinear dynamic system generates new data. It can be used to describe the dynamic fluctuation of speech signals in response to various emotions. To process SE and its statistics, as well as MFCC, and calculate the probability that they belong to one of six emotions, the support vector machine is used. The spoken English recognition algorithm described in this paper has obvious performance improvements in many indicators, according to the experimental evaluation.

## 1. Introduction

At present, computer-aided evaluation system has gradually become one of the research hot spots [1]. Especially in large-scale examinations, it has begun to gradually replace teachers, which will become a major change in education. With the development of speech recognition technology, the speech evaluation system is also developing gradually. In spoken English recognition, the original phonetic training data is not accompanied by any language information. Therefore, the language information itself must be estimated during training [2, 3]. The method of discovering language categories is valuable for their potential application and theoretical insight. Therefore, spoken English has always been one of the main concerns in linguistics, rhetoric, discourse analysis, and corpus linguistics [4]. People study spoken English from many angles, including the description of the linguistic features of spoken English.

There are some features of pronunciation that Chinese English learners overlook, and there is no lack of neglect of some features in spoken English, which makes it the bottleneck for improving their spoken English level. It is uncommon to record the candidates' answers on the spot in a traditional oral exam [5, 6]. This makes it impossible to recreate the examinee's examination situation to other teachers or classmates at the time, as well as to comment on the examinee's answers at the time, and other candidates are unable to benefit from the examinee's experience and lessons [7]. For oral question-and-answer types, there are two methods of scoring: one is based on pronunciation, and the other is based on text. Pure speech scoring focuses on acoustic characteristics like pronunciation, frequency, and rhythm [8, 9]. This method, on the other hand, can be effective in limiting the types of speaking content. However, it is a little out of reach for open questions. The development of a multifeature fusion evaluation algorithm for spoken English based on speech recognition, natural language processing, and data fusion will pave the way for the development of an automatic spoken English evaluation system that can interact with users [10]. The learning efficiency of spoken English will be greatly improved by systematically imitating people's ways of thinking, evaluating users' spoken English, and providing suggestions.

This paper will look at audio processing, speech recognition, and intelligent English correction technology and design and implement a multifeature intelligent automatic correction model based on data from a college language training system. The automatic correction model aims to address the issue of automatic correction of open oral English questions, reduce teacher correction pressure, and provide effective oral English learning guidance for students.

## 2. Related Work

Spoken English is a language feature system that meets the needs of English users' oral communication. The immediacy, immediacy, and improvisation of oral communication determine that spoken English has its distinctive and unique linguistic features. In [11] through the research on the application of automatic speech recognition system to nonnative language speech training, it is pointed out that if appropriate methods are used and mispronunciation detection is added, the evaluation system can provide the same evaluation results as human experts. In [12] pronunciation evaluation is divided into three parts: similarity of pronunciation content, similarity of pronunciation and intonation, and score adjustment based on nonlinear attenuation. Literature [13] proposed an algorithm to find similar speech sequences. This algorithm is also based on dynamic time planning algorithm. Compared with [14], the starting point and ending point are predefined, and then the similar phonetic sequences are found to be different in predefined areas. Literature [15] calculates all possible similarities and then determines the boundary of subsequences according to the similarity changes between adjacent elements. Literature [16] lists the challenges to be faced by modeling the simulated human score from the process and result levels and points out that it is impossible to model the evaluation process comprehensively in the field of speech features and speech recognition at present. In [17] evaluating what the interlocutor said at the phoneme level has the advantages that it can accurately locate where the speaker's pronunciation is wrong, evaluate the similarity between what the speaker said and the target pronunciation, and find the systematic difference by comparing it with the standard phonetic database.

Literature [18] divides pronunciation evaluation into three parts: similarity of pronunciation content, similarity of pronunciation and intonation, and score adjustment based on nonlinear attenuation. Based on the technology of speech recognition and accent adaptation based on hidden Markov model, this paper studies the accuracy and fluency of phoneme pronunciation, gives the pronunciation quality score at phoneme level, and further obtains the score result of the whole sentence. In [19] in the research of the same automatic composition correction system, it is pointed out that some composition correction systems have great deficiencies in content-based feedback and have certain limitations, such as being unable to identify whether students' compositions are irrelevant. Literature [20] studies an improved speech recognition method based on deep learning [21], which effectively improves the relevance of human-machine scoring in the spoken language scoring system based on speech recognition. Literature [22] discusses the significance of automatic correction system for online correction of English compositions, because automatic correction of compositions can not only reduce the burden of teachers, but also respond quickly, and the correction time is in seconds. Moreover, the automatic correction system has a profound influence on the role orientation of students and teachers and the teaching mode. Literature [23] expounds a working principle of automatic correction system. In [24] using the traditional machine learning model, Hidden Markov Model, to process audio files, directly extract sound features from audio, and then combine them with the rules of oral English scoring, an intelligent scoring system for oral English test was developed. Their research is very respectable. Not only is the model training of machine learning complicated, but the processing of audio files is extremely difficult. Voiceprint recognition has always been an important and difficult point for many scientists. Finding the rules of human voice has a very far-reaching impact on speech recognition and machine translation.

## 3. Research Method

### 3.1. Design of Intelligent Correction System for Spoken English

The connection mode of the campus LAN (local area network) is adopted between the student computer and the server. Because, after the exam, the student computer needs to upload a large number of audio data files to the server, it is required to get the maximum network transmission bandwidth between the student computer and the server as much as possible to reduce the delay caused by network transmission. Therefore, adopting the campus LAN can ensure the smooth progress of the examination process.

The application of this system will greatly improve the efficiency of oral examination, reduce the inequality caused by human factors in the course of oral examination, reproduce the candidates' answers at that time, improve students' oral English level, reduce the work intensity of teachers, and increase the safety and reliability of the examination.

**Figure 1:**
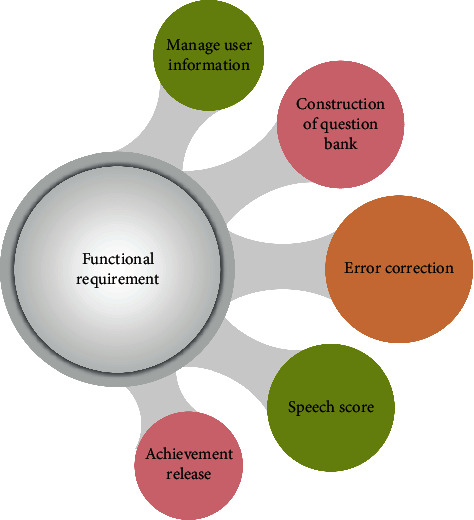
Functional requirements of the system.

The main function of the system is the learning and testing of spoken English. This requires the system administrator to set the learning mode or testing mode. The functional requirements of the system are shown in Figure 1.

In learning mode, the user first enters the system through a visual window, then selects the appropriate question bank, activates the demonstration voice, and follows and practices the instructions. The voice prompt and error correction functions are currently available. When a user enters the system in test mode, the system selects questions at random to ensure that the exam is fair. The voice prompt and error correction functions are currently disabled. The examinee's login, checking the examinee's information, auditioning before the exam, random selection of test questions, answering recording, and uploading the answering recording after the oral exam are the main functions of the oral exam. On the student test machine, the recording is completed, then packaged, and uploaded to the server. The server creates a corresponding folder based on the student's student ID and stores the received files in the folder for teachers to review and use after receiving the uploaded files.

The statistical subsystem is the feedback of candidates' achievements to teachers. Its main job is to help teachers make statistics after obtaining a large number of test scores. Teachers will refer to the statistical results to better guide students to complete their studies and exams, so that they can achieve better results in future exams.

Computer-aided test system can help teachers or teaching administrators to design tests and generate test papers, implement tests under certain conditions, analyze tests, manage scores, and provide reports. Therefore, there are reasons to believe that we can design a computer-aided testing system specifically for spoken English testing.

**Figure 2:**
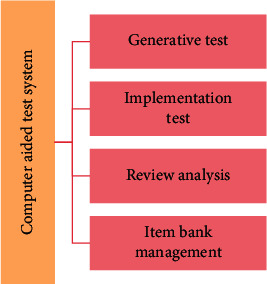
Functional module structure of computer-aided test system.

A complete computer-aided test system not only has a question bank, but also has the functions of testing, evaluation, and analysis. The specific functional module structure is shown in Figure 2.

The computer can upload the results of students' answers to the server, and the server automatically judges objective questions such as multiple-choice and true-false questions, assigns scores based on whether they are correct or not, and records and saves the evaluation results of each student's test (generally including the question number, true or false information, waiting time for answers, and so on) for test analysis. For example, in the case of a balanced distribution of test difficulty, the majority of candidates can correctly answer the test's questions, while only those with poor academic performance can correctly answer fewer questions, indicating that this period of teaching was successful. For another example, if the candidates with excellent grades did not get it right but the candidates with poor grades did, this demonstrates that the topic cannot distinguish between excellent and poor students.

When the purpose of the test is to classify students and judge their level and ability, always hope that the test is fair and accurate. According to the requirements of educational evaluation, the test should have two necessary characteristics: evidence (validity) and reliability (reliability). Test paper analysis is the analysis of the reliability and validity of the test paper. The results of the analysis can reflect whether the test really checks the students' knowledge level and ability and whether the teaching process is successful or not. That is to say, it can be judged whether the test results can be used as the basis for evaluation.

### 3.2. Oral English Evaluation Algorithm Based on Multifeature Fusion

#### 3.2.1. Feature Extraction

To evaluate the quality of a person's spoken English, usually give an overall evaluation first; for example, this person's spoken English is very good; then predict that the person's pronunciation is accurate and his intonation is well mastered, but his continuous reading is not good enough, and so on. The purpose of this study is to evaluate users' spoken English by computer and give the evaluation results. This requires a comprehensive evaluation algorithm, and an evaluation algorithm based on multifeature fusion is given in this paper.

This paper's proposed multifeature fusion evaluation algorithm is based on existing single-feature evaluation algorithms. At the very least, a multifeature comprehensive evaluation method should have the following characteristics: scalability, which means that the features chosen and the number of features chosen will have little impact on the system, and it should be simple to add and remove features. It has nothing to do with a single feature's evaluation algorithm; that is, changing the evaluation algorithm of a single feature has no effect on the system when the form of the evaluation result of a single feature remains unchanged.

**Figure 3:**
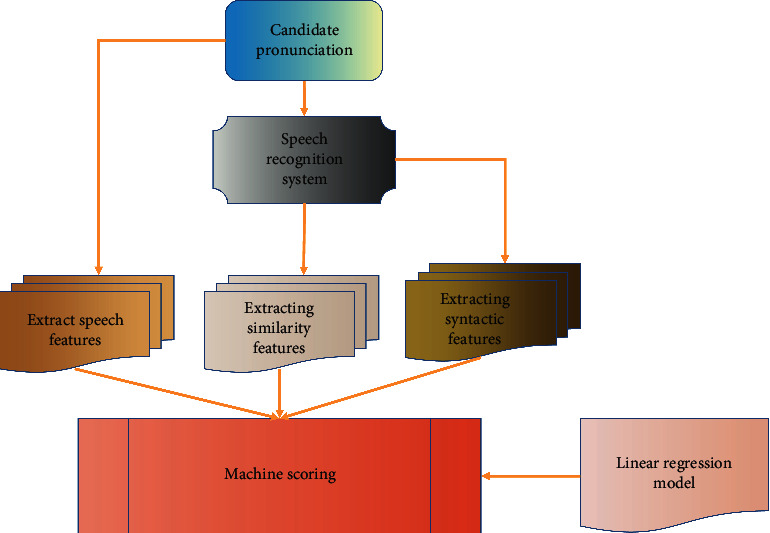
Scoring system framework.

As shown in Figure 3, the whole scoring system includes three parts: speech recognition, feature extraction, and linear regression.

The similarity features and syntactic features in feature extraction are extracted from the text after speech recognition, and the similarity features need to be compared with reference answers. Syntactic features are mainly used to measure the grammatical level of candidates' answers. The phonetic features, including pronunciation confidence and speaking fluency, are used to judge the accuracy of pronunciation and speaking fluency of candidates. These features are extracted from the preferred recognition results of speech recognition. Finally, score multiple features by using the trained linear regression model.

Firstly, multiple features are evaluated separately, and multiple evaluation results are obtained. Then quantify these evaluation results, the quantization standard is obtained through systematic training in advance, and the quantization result is *S*_1_, *S*_2_,…*S*_*n*_. Then, the weighted average of these quantized results is carried out to obtain the quantization result of comprehensive evaluation:

(1) $$\begin{array}{c} S=\sum_{i=1}^{n}a_{i}S_{i}, \end{array}$$

where *a*_*i*_ is the weight of each feature, ∑_*i*=1_^*n*^*a*_*i*_=1; *S*_*i*_ is the quantified value of each evaluation result.

Finally, based on the quantitative results, according to certain standards, the evaluation results are given. The evaluation results are divided into four grades, namely, “bad,” “normal,” “good,” and “excellent”.

SE (sample entropy) is a new method for measuring the complexity of time series, which is an improved statistic that does not count self-matching for the approximate algorithm.

It is defined as the conditional probability that the data vector will continue to maintain its similarity when it increases from *m* dimension to *m*+1 dimension, and the SE of the original data with *N* points is expressed as

(2) $$\begin{array}{c} \text{SampEn}\left(m,r,N\right)=-\ln\frac{B^{m+1}\left(r\right)}{B^{m}\left(r\right)},\\ SampEn\left(m,r,N\right)=-\ln\frac{B^{m+1}\left(r\right)}{B^{m}\left(r\right)}. \end{array}$$

Traditional MFCC (Mel frequency cepstrum coefficient) only reflects the static features of speech signals, while SE can describe the dynamic changes of speech signals. The two features have different roles in distinguishing emotions. The two features of SE and MFCC are input into support vector machine, respectively, and the probabilities that they belong to six emotions are calculated. Then, the recognition results are obtained by combining addition rules and multiplication rules. This section concludes two kinds of fusion rules.

This paper uses two different fusion rules:

The addition rule is
  (3) $$\begin{array}{c} F\left[P\left(\omega_{i}|f_{1}\right),\ldots ,P\left(\omega_{i}|f_{R}\right)\right]=\sum_{j=1}^{R}P\left(\omega_{i}|f_{j}\right). \end{array}$$
The multiplication rule is
  (4) $$\begin{array}{c} F\left[P\left(\omega_{i}|f_{1}\right),\ldots ,P\left(\omega_{i}|f_{R}\right)\right]=\prod_{j=1}^{R}P\left(\omega_{i}|f_{j}\right). \end{array}$$

The algorithm steps of speech emotion recognition based on multifeature fusion of SE and MFCC are as follows:

Reading emotional speech signals and preemphasizing, framing, and windowing, with the frame length of 256 and the frame shift function of 128 as Hamming window;

Preprocessed speech signals of each frame are original data *x*(1), *x*(2),…, *x*(*N*), and this data is composed into a group of *m*-dimensional vectors in a continuous order:

(5) $$\begin{array}{c} x\left(i\right)=\left[x\left(i\right),x\left(i+1\right),x\left(i+m-1\right)\right],\;i=1∼N-m+1. \end{array}$$

Average *B*_*i*_^*m*^(*r*):

(6) $$\begin{array}{c} B^{m}\left(r\right)=\frac{1}{N-m+1}\sum_{i=1}^{N-m+1}B_{i}^{m}\left(r\right). \end{array}$$

According to the principle of addition and multiplication rules, the posterior probabilities of the two features are fused, and then the recognition rates of six emotions are calculated, respectively.

#### 3.2.2. Semantic Discovery

When communicating in English, many Chinese college students rarely or never include nonverbal factors, such as lack of communicative competence in expression, body movements, and tone. Spoken English is the first step toward understanding a language and culture that is not one's own. When nonlanguage is combined with spoken English, one can gain a more comprehensive and in-depth understanding of the language, which will help students improve their practical ability to use English. Using dynamic programming technology, the semantic discovery algorithm detects the repeated parts of acoustic speech signals. In general, finding the shortest distance between sound input and a set of templates is used to recognize all duplicate parts. Local comparison [25] is used in this paper to reveal the repeated subparts of two acoustic signals using the cumulative quality scoring mechanism.

Input each spoken English as an acoustic feature vector *U*_*m*_, and compare it with the stored feature vector *V*_*n*_. The calculation method of cosine similarity is shown in formula (7).

(7) $$\begin{array}{c} D_{ij}=\frac{U_{i}\cdot V_{j}}{\left\|U_{i}\right\|\cdot \left\|V_{j}\right\|}. \end{array}$$

For convenience of explanation, based on the above assumptions, in order to evaluate the reliability of system IP (indistinguishable phoneme) recognition, two relative measures are introduced, namely, the correct recognition rate *Qr* and the false recognition rate *e*_*i*_^*j*^. The following are their exact definitions:

(8) $$\begin{array}{c} r=\frac{N_{\text{right}}}{\left(N_{\text{right}}+N_{\text{error}}\right)},\\ e_{i}^{j}=\frac{N_{c}\left(i,j\right)}{n_{t}\left(i\right)}, \end{array}$$

where *N*_right_ is the number of phonemes correctly recognized by the system; *N*_error_ is the number of phonemes wrongly recognized by the system.

The normalization of input features refers to measuring the input feature values on the same standard. For example, the range of the output results of the grading network for vocabulary and grammar is [0.1]. The result of teacher's manual grading is 10 points. Therefore, it is necessary to normalize the teacher's manual score and the oral feature score mentioned earlier in this paper, so that the range of input features is uniform when training the multifeature correction model later. And after normalization, it can show candidates all the characteristics of spoken English. Namely:

(9) $$\begin{array}{c} x_{i}^{p^{*}}=\frac{x_{i}^{p}-x_{i}^{\min}}{x_{i}^{\max}-x_{i}^{\min}}. \end{array}$$

In many spoken language scoring systems studied by predecessors, the scoring model is established by linear regression, which is a classic machine learning algorithm and can fit and predict data with linear characteristics. This paper will also use the method of linear regression to establish the scoring model. The definition of linear regression is as follows:

(10) $$\begin{array}{c} h_{\theta }\left(x\right)=\theta_{0}+\theta_{1}x_{1}+\cdots +\theta_{n}x_{n}, \end{array}$$

where *n* is the number of input features, and in this paper, *n*=5. A linear regression model was also built using TensorFlow. Similarly, 80% of data sets are used as training sets and 20% as test sets.

## 4. Results Analysis and Discussion

### 4.1. Multifeature Recognition Results

**Figure 4:**
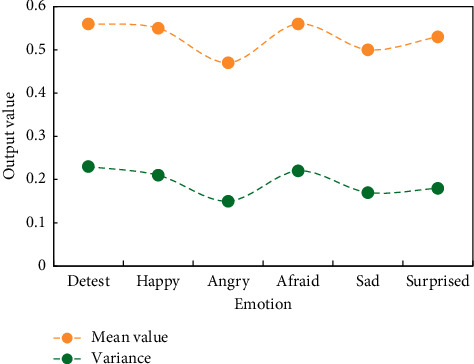
SE statistics of six kinds of emotions.

**Figure 5:**
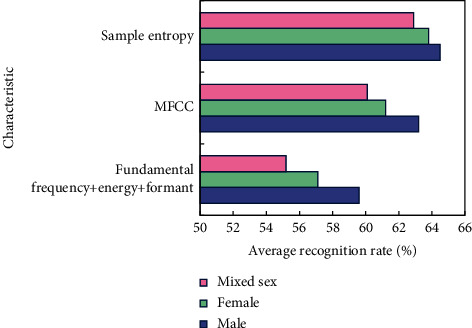
Average recognition rate of three kinds of features and their statistics.

In the simulation, all speech samples are divided into male, female, and mixed voice, and SE, fundamental frequency, energy, formant, and MFCC are extracted and their statistics are calculated. The mean and variance of SE are shown in Figure 4, and the average recognition rate of the three types of features and their statistics is shown in Figure 5.

Students have a strong desire for knowledge about the content of teachers' lectures, and their learning mentality is positive. Then, in the process of oral English practice, they need to be very careful and serious and will actively work to solve these problems and, at the same time, use a variety of oral English practice methods.

Their oral English ability is also different, but they are all studying hard, but their understanding of oral English can be summarized as repeated practice, and the content is mainly arranged by textbooks and teachers, while some English teachers are only teaching and emphasizing how students practice spoken English repeatedly; that is, they spend a lot of time repeatedly practicing reading every English word and sentence.

**Figure 6:**
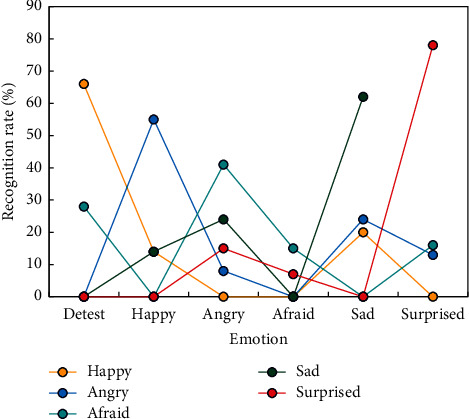
SE and its statistics on six kinds of emotion recognition rates.

Figure 5 shows that the recognition rate of SE as a feature parameter is higher in male, female, and mixed voice samples than the other two feature parameters, with male voice samples having the highest recognition rate of 64.5 percent.  Figure 6 shows the recognition results of six emotions of mixed male and female voice when SE is the feature parameter.

As can be seen from Figure 6, when SE and its statistics are used as feature parameters, the recognition rate of happy and surprised emotions is as high as 80%, the resolution of SE to happy and surprised emotions is high, and the emerging new information has a good distinction with other emotions.

**Figure 7:**
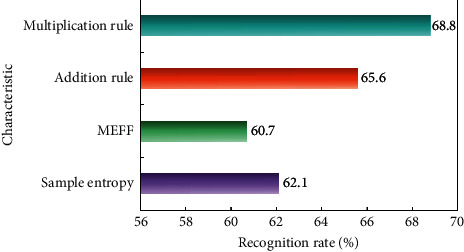
Average recognition rate of SE and MFCC and their fusion.

Using the addition rule and multiplication rule in the previous section to fuse SE sum, respectively, the recognition result is shown in Figure 7.

**Figure 8:**
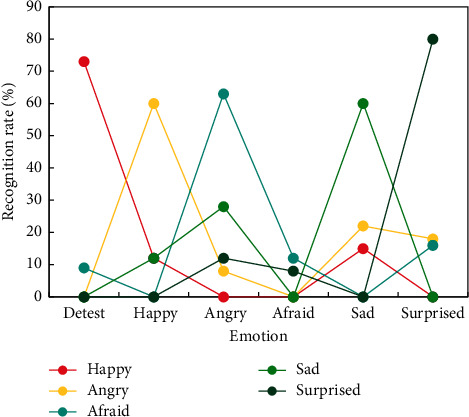
Six emotion recognition rates based on the fusion of SE and MFCC multiplication rules.

From Figure 7, it can be seen that after SE and MFCC are merged, the recognition rate is obviously improved, and the highest recognition rate of multiplication rules is 68.8%. SE and MFCC have different emphasis on distinguishing emotions, and the recognition effect after fusion is ideal. The recognition rate of six emotions fused by multiplication rules is shown in Figure 8.

The recognition rate of happy emotions decreases after SE and MFCC are merged according to the multiplication rule, as the error rates of estimated probabilities of SE and MFCC accumulate, reducing the recognition rate of happy emotions. Fear and aversion emotion recognition rates improve dramatically after merging, and the average recognition rate of six emotions rises to 66.3 percent, up 3.9 percent from SE before merging. Various cultures found in the United States or English should be combined with a lot of oral English teaching. Because of the various users and environments, English will take on a variety of roles and meanings, particularly given the long-standing lifestyle differences between Westerners and Chinese. As a result, in the process of teaching oral English, teachers should not only pay attention to the pronunciation and grammatical accuracy of English words and phrases, but also teach students to understand the communication environment of characters and the relationships between characters in oral English, as well as analyzing the different meanings expressed by different words and phrases in different situations, because the change of tone directly affects the meanings expressed by different words and phrases in different situations.

### 4.2. Analysis of Intelligent Correction System for Spoken English

Creating a good atmosphere can help students get into the specific situation of the text faster and better and help students imagine abstract written words as concrete, vivid, and vivid pictures in their minds, so as to stimulate emotional resonance, enhance students' language perception ability, and finally let students enter the state of “reading aloud with affection.” Reading forms can be arranged according to the classroom time, such as reading competitions among individuals, reading competitions among groups, reading by roles, etc. Individual reading forms are helpful for teachers to give targeted guidance and comment on students' reading effects, help students to correct shortcomings, and thus improve students' level of “emotional reading.”

**Figure 9:**
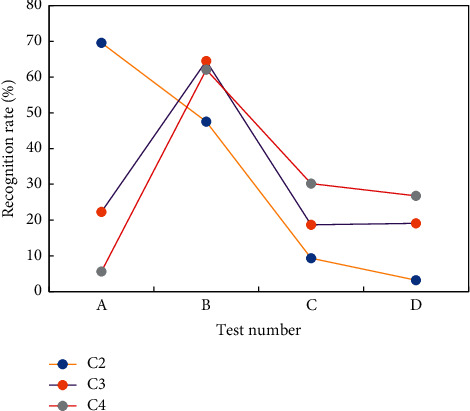
Comparison of open test results.

The training corpus C1 contains 1039 native natural oral recordings and scripts, while the test corpus C2 contains 128 IP clusters from native natural pronunciation recordings. Simultaneously, in order to complete the robustness testing, the author chose two students, one of whom graduated with a major in English and the other with a major in computer science. They have different levels of oral proficiency, so their recordings of the same corpus are labeled C3 and C4, respectively. The results of voice evaluation for C2, C3, and C4 are shown in Figure 9.

These results show that our evaluation model can get reliable and robust evaluation results with the average phoneme recognition rate of the system.

In college, students are taught English orally. Schools have not paid much attention to English. Intensive reading and listening are two types of college English courses offered by many universities. While most listening courses are conducted in a centralized manner, which is limited by factors such as teachers, class size, and so on, the accuracy of the course accounts for nearly 3/4 of the total class hours. Furthermore, most universities do not include an oral exam in their English exams, resulting in students paying little attention to oral English learning.

The survey shows that 90% of teachers think that they mainly play the role of interpreter and demonstrator in English class, and 70% of teachers think that their role is mainly to guide students how to learn foreign languages and how to cultivate their foreign language learning ability. It can be seen that most English teachers can clearly understand how to effectively carry out teaching but rarely do this in actual teaching.

**Figure 10:**
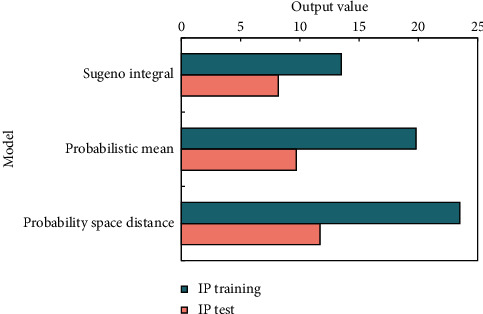
IP training and test results.

Figure 10 shows the size of training or test set; it reflects the performance of training or testing. The larger the performance, the better the performance.

From the above data, the integration method has the best stability, followed by the probability space distance method, and the probability mean method is the worst. In terms of performance, the method based on probability space distance is the best, followed by probability mean method, and the method based on Sugeno integral is the worst.

Teachers should create a positive learning environment for students, plan rich classroom activities, actively guide students to look at pictures and talk, intensify oral practice, and provide students with plenty of practical practice opportunities when teaching oral English. Their oral English level can only be improved to some extent through continued practice and practice. In order to improve one's language communication level, the process must be undertaken step by step, from easy to difficult, simple to tedious, and success cannot be rushed. Mechanical training can be done at the start of the training to gradually increase the level of fluent expression. Different ways of thinking exist in Chinese and foreign cultures, resulting in differences in language expression. The verbs in Chinese sentences are usually centered and spread horizontally, forming a flowing shape structure. The structure of Westerners' sentences is generally centered on subjects or predicates, and it is more complicated. As a result, students should use correct logical thinking to express themselves in the process of language expression, gain a deeper understanding of western culture, and improve the flexibility and smoothness of their oral expression.

Visual effects can make students more aware of the feelings attached to English that they have heard. In the practical use of English, when people communicate in English, they not only rely on their ears to listen, but also want to really understand what the other party wants to express, and they should combine their visual senses. In view of this, teachers can choose English movies that meet the nonverbal communication factors for teaching. First, let students learn the nonverbal factors in the movies, because speaking English alone cannot effectively improve students' oral ability.

## 5. Conclusion

In college English classes, oral English instruction is a weak point. There are numerous issues that arise during the teaching process, such as a lack of a language environment, fewer class hours, and so on, and students' application ability is low. The analysis and design of the intelligent correction system for spoken English, as well as the specific implementation method, are detailed in this paper. The system greatly improves the efficiency of oral English tests while also reducing the difficulty of organizing them and teachers' workload. When SE is used alone as an emotion recognition feature, it outperforms MFCC and fundamental frequency. Finally, the MFCC and SE fusion recognition results are examined, and the fusion recognition rate is improved. Experiments show that the speech evaluation system presented in this paper is more in tune with people's subjective feelings and that the evaluation results can reflect the subjects' pronunciation level.

Due to the author's limited knowledge, there are still many flaws in the system's operation, and there are numerous areas for improvement and further research, such as expanding the question bank, improving system security, increasing system efficiency, and designing an analysis system for the data gathered during the examination process, among other things. In future research, the author will investigate and analyze the aforementioned issues in greater depth, with the goal of improving the system's functionality.

## Data Availability

The data used to support the findings of this study are included within the article.
